# Efferocytosis dysfunction in CXCL4-induced M4 macrophages: phenotypic insights in systemic sclerosis *in vitro* and *in vivo*

**DOI:** 10.3389/fimmu.2024.1468821

**Published:** 2024-10-11

**Authors:** Erwan Le Tallec, Nessrine Bellamri, Marie Lelong, Claudie Morzadec, Quentin Frenger, Alice Ballerie, Claire Cazalets, Alain Lescoat, Frédéric Gros, Valérie Lecureur

**Affiliations:** ^1^ INSERM, EHESP, IRSET (Institut de Recherche en Santé, Environnement et Travail) – UMR_S 1085, Univ Rennes, Rennes, France; ^2^ Department of Internal Medicine and Clinical Immunology, Pontchaillou Hospital, Rennes, France; ^3^ INSERM UMR - S1109, Université de Strasbourg, Strasbourg, France; ^4^ Faculty of Life Sciences, Université de Strasbourg, Strasbourg, France

**Keywords:** CXCL4, efferocytosis, fibrosis, macrophage, phagocytosis, systemic sclerosis

## Abstract

**Introduction:**

Systemic sclerosis (SSc) is an autoimmune disease characterized by antinuclear antibody production, which has been linked to an excess of apoptotic cells, normally eliminated by macrophages through efferocytosis. Additionally, circulating levels of CXCL4, a novel SSc biomarker, correlate with more severe fibrotic manifestations of the disease. Considering the defective efferocytosis of macrophages in SSc and the CXCL4-related M4 macrophage phenotype, we hypothesized that CXCL4 could be involved in the alteration of phagocytic functions of macrophages in SSc, including LC3-associated phagocytosis (LAP), another phagocytic process requiring autophagy proteins and contributing to immune silencing.

**Methods:**

In this study, CXCL4 levels were measured by ELISA *in vitro* in the serum of SSc patients, and also *in vivo* in the serum and lungs of C57BL/6J SSc mice induced by intradermal injections of hypochloric acid (HOCl) or Bleomycin (BLM), with evaluation of M4 markers. Circulating monocytes from healthy donors were also differentiated *in vitro* into M4 monocytes-derived macrophages (MDMs) in the presence of recombinant CXCL4. In M4-MDMs, phagocytosis of fluorescent beads and expression level of efferocytic receptors were evaluated by flow cytometry *in vitro*, while efferocytosis of pHrodo-stained apoptotic Jurkat cells was evaluated by real-time fluorescence microscopy. LAP quantification was made by fluorescence microscopy in M4-MDMs exposed to IgG-coated beads as well as apoptotic Jurkat cells.

**Results:**

Our results demonstrated that efferocytosis was significantly reduced in M0-MDMs from healthy donors exposed to the CXCL4-rich plasma of SSc patients. *In vivo, CXCL4* expression was increased in the lungs of both SSc-mouse models, along with elevated M4 markers, while efferocytosis of BLM-mice alveolar macrophages was decreased. *In vitro*, M4-MDMs exhibited reduced efferocytosis compared to M0-MDMs, notably attributable to lower CD36 receptor expression and impaired phagocytosis capacities, despite enhanced LAP. Autophagic gene expression was increased both *in vitro* in SSc MDMs and *in vivo* in BLM mice, thus acting as a potential compensatory mechanism.

**Discussion:**

Altogether, our results support the role of CXCL4 on the impaired efferocytosis capacities of human macrophages from SSc patients and in SSc mice.

## 1 Introduction

Systemic sclerosis (SSc) is a chronic and irreversible systemic autoimmune disorder characterized by progressive and irreversible skin and lung fibrosis, associated with microvascular dysfunction. In SSc, antinuclear autoantibodies may, in part, arise from a defect of the efferocytosis function of macrophages (1). This defect impairs the clearance of apoptotic cells containing intracellular autoantigens and danger signals, participating in the onset of systemic autoimmunity (2). LC3-associated phagocytosis (LAP) is another macrophage mechanism involved in the clearance of apoptotic cells, which utilizes the autophagy machinery to link LC3 to phagosomal and autophagic vesicle membranes, thus contributing to the maturation of LC3-II vesicles (LAPosomes) and enhancing lysosomal fusion (3). While there is currently limited data on autophagy in SSc, LAP has never been studied in this disease. We previously demonstrated an impaired efferocytosis in macrophages from SSc patients (4). Macrophages play a pivotal role in SSc’s pathogenesis and participate in the progression of the fibrotic manifestations of the disease (5). Macrophages can exhibit diverse and heterogeneous phenotypes or polarization states depending on the microenvironment (6). While macrophages are commonly categorized as classically activated M1 pro-inflammatory macrophages and alternatively activated M2 macrophages, some of which display pro-fibrotic properties (7), recent research supports the idea of a mixed macrophage signature in SSc beyond this binary classification (8–10). Moreover, macrophage efferocytosis is modulated by their environment (11), and we have previously observed varying efferocytosis capacities in SSc macrophages derived from monocytes, suggesting the influence of SSc serum on monocytes before *in vitro* differentiation (4). Notably, elevated levels of the platelet chemokine CXCL4, a novel biomarker of systemic sclerosis (12), have been linked to the fibrotic complications of the disease (13). Additionally, CXCL4 has been shown to inhibit monocyte apoptosis (14) and to induce their differentiation into a distinct and specific M4 macrophage phenotype (15). Therefore, we hypothesized that the overexpression of CXCL4 in SSc may induce a M4 phenotype of macrophages with defective efferocytosis.

## 2 Materials and methods

### 2.1 Chemicals and reagents

Human recombinant cytokine CXCL4 was purchased from Peprotech (Neuilly sur Seine, France). Human recombinant GM-CSF and M-CSF were obtained from Sanofi-Aventis (Montrouge, France) and Miltenyi Biotec SAS (Paris, France), respectively. Bleomycin sulfate (BLM), camptothecin, and cytochalasin D (CytoD) were purchased from Sigma-Aldrich (St-Quentin Fallavier, France).

### 2.2 Human blood samples

Patients with SSc satisfied the 2013 ACR/EULAR classification criteria for SSc (16), and their clinical presentation was defined according to LeRoy et al. (17). They were consecutively included after written informed consent. This study was approved by the local ethics committees (Committees for Protection of Persons (CPP) Ouest-V France, CPP approval N°: 2019-A02611-56). Information regarding patients is resumed in **Supplementary Tables 1** and **2**. Blood samples (PBMCs and sera) were collected from SSc patients at the Department of Internal Medicine and Clinical Immunology, Rennes Hospital, France, and those from healthy donors (HD) from Etablissement Français du Sang, Rennes, France.

### 2.3 Primary cultures of human macrophage

Human macrophages were differentiated from peripheral blood monocytes. Buffy coats of healthy donors were provided by Etablissement Français du Sang (Rennes, France) and obtained after the written consent for the use of blood samples for experimental research. Peripheral blood mononuclear cells were obtained from blood buffy coats of HD or the blood of SSc patients through Ficoll gradient centrifugation. The monocytes, selected after a 1h adhesion step, were differentiated into macrophages (M0) during 6 days in RPMI 1640 medium GlutaMAX (Gibco, Life Technologies), containing 10% heat-inactivated fetal bovine serum (FBS, Lonza, Levallois-Perret, France), 2 mM L-glutamine, 20 IU/mL penicillin, 20 μg/mL streptomycin (ThermoFisher Scientific, Courtaboeuf, France) and 50 ng/ml of M-CSF or 400 IU/ml GM-CSF. The medium was replaced on day 4 and day 6. From day -6 to day 8, cells were maintained in a fresh medium containing 5% FBS either without GM-CSF (GM-M0) either 10 ng/ml M-CSF (M-M0) or supplemented with 1 µM CXCL4 for M4 polarization.

### 2.4 Mouse model of BLM-induced systemic sclerosis

Male C57BL/6J mice weighing between 18 and 20 gr, used at 8 weeks of age, were acquired from Janvier Labs (Le Genest Saint Isle, France). The animals were all housed in similar autoclaved cages and fed food and water with identical housing conditions. They were maintained under a 12/12h light/dark cycle, with controlled room temperature and humidity. Experimental scleroderma-associated ILD was induced by daily intradermal injections of BLM solution (0.4 mg/kg in 100 µl) into the shaved back of mice (5 days a week) for 4 weeks. Serum and tissue biopsies were collected at the end of the experiment at day 28. Mice were randomly divided into 2 groups: intradermal injections of NaCl (n = 6), and intradermal injections of BLM dissolved in NaCl (n = 6). The number of groups and mice per group was pre-calculated depending on statistical power considerations based on the expected results on skin involvement and data from the literature. Animal studies were reviewed and approved by the Committee on the Ethics of Animal Experiments under the French Ministry of Higher Education and Research (#17011-2018100812449655). The study was carried out in strict accordance with the recommendations in the Guide for the Care and Use of Laboratory Animals, EEC Council Directive 2010/63/EU.

### 2.5 Mouse model of HOCl-induced systemic sclerosis

Female C57BL/6J mice weighing between 18 and 20 gr, used at 8 weeks of age and purchased from Janvier Labs (Le Genest Saint Isle, France) were randomly divided into 2 groups: daily intradermal injections of 100 µl of PBS (n = 9) or HOCl (n = 8, one loss due to biopsy sample at week 3) as previously described (18). Serum and tissue biopsies were collected at the end of the experiment at day 42. Animal studies were reviewed and approved by the Committee on the Ethics of Animal Experiments under the French Ministry of Higher Education and Research (#17011–2018100812449655).

### 2.6 Phagocytosis assays by flux cytometry

MDMs were treated for 1 h with 5 μM of cytochalasin D (CytoD), an actin polymerization inhibitor that prevents cytoskeletal remodeling, and serves as a negative control for phagocytosis. The cells were incubated in the presence of fluorescent latex microspheres (ratio 10:1) (Fluoresbrite™ Plain YG 1.0 Micron Microsphere, Polysciences, Warrington, USA) for 45 min at 37°C or at 4°C (negative control). After several washing with PBS to eliminate the non-phagocytosed beads, MDMs were detached in the presence of Accutase^®^ cell detachment solution. The fluorescence emitted at 525 nm by the cells having achieved phagocytosis was quantified by flow cytometry on an LSR II cytometer with FACSDiva software (BD Biosciences). The results were expressed in % of phagocytosis, calculated as follows: % cells fluorescent (37°C) - % cells fluorescent (4°C).

### 2.7 Induction of apoptosis in Jurkat cells and quantification of efferocytosis in human and lung murine macrophages

#### 2.7.1 Induction of apoptosis

The human Jurkat T CD4 lymphocyte cell line was cultured in RPMI 1640 Glutamax medium supplemented with 10% heat-inactivated FCS and antibiotics. Apoptosis induced in Jurkat cells (Japo) through exposure to 10 μM camptothecin for 4 h, was confirmed with FITC-Annexin V/iodide propidium staining by flow cytometry on an LSR II cytometer as previously described (4).

#### 2.7.2 Quantification of efferocytosis in human macrophages

Apoptotic Jurkat cells (Japo) were stained for 15 min with 250 ng/ml pHrodo (IncuCyte^®^ pHrodo^®^ Red Cell Labeling Kit, Sartorius, Ann Arbor, USA), washed and added to MDMs plated in 96-well-tissue culture plates, in 10:1 ratio (apoptotic cells/MDM) at 37^°^C in a 5% CO_2_ humidified incubator. Engulfment efficiency of apoptotic cells by MDMs was quantified by real-time fluorescence microscopy (IncuCyte^®^ live-cells Analysis system, Sartorius), measuring total integrated red intensity (ex:560nm/em:585nm) of labeled Jurkat cells when entering the acidic phagosome every 15 min for 3 h. The average fluorescence intensity (expressed as RCU x µm^2^/image) at 90 min was used to compare the level of efferocytosis between the different conditions.

#### 2.7.3 Quantification of efferocytosis in BLM mouse model

After staining for 15 min with 100 ng/ml CellTrace™CFSE (Invitrogen, ThermoFisher scientific), Jurkat cells were washed and then exposed to 10 µM of campothecin for 4h to induce apoptosis. An oropharyngeal instillation of 5.10^6^ cells/50 µl CFSE-stained apoptotic cells was then performed in anesthetized mice. The bronchoalveolar lavages (BAL) were performed 2 h after the instillation of apoptotic cells, to evaluate the efferocytosis capacities of alveolar macrophages (AM) (38). Cells from BAL were re-suspended in PBS supplemented with 2% FCS solution containing Fc-block and then stained with mouse anti-CD11b-PE-Cy7 (BD Biosciences, San Jose, CA, USA) and anti-Gr1-V450 (eBiosciences SAS, Paris) antibodies. The efferocytosis capacities were measured in AM (Gr1^Int^ and CD11b^Int^). The engulfment efficiency was measured by flow cytometry.

### 2.8 LC3-associated phagocytosis quantitative analysis of immunofluorescence images

#### 2.8.1 Labeling of human Jurkat lymphocytes by PKH67

Jurkat cells were labeled for 5 min with the fluorescent probe PKH67 (Sigma-Aldrich), exhibiting a high affinity for membrane lipids. After washing with RPMI medium, apoptosis of PKH67-stained Jurkat cells (Japo PKH67+) was induced by treatment with camptothecin (see paragraph 2.6.1).

#### 2.8.2 Induction of efferocytosis

Macrophage plated in Merck Millipore Millicell™ EZ Slides 8-well glass (Thermo Fisher Scientific, Courtaboeuf, France) were exposed to apoptotic Jurkat cells stained by PKH67 (Sigma Aldrich, Saint Quentin Fallavier, France) in 10:1 ratio for 45 min at 37°C in a 5% CO2 humidified incubator.

#### 2.8.3 LAP immunostaining

After several washing MDMs with PBS to eliminate the non-phagocytosed Jurkat, MDMs were fixed with a solution of 4% paraformaldehyde (Santa Cruz Biotechnology, Dallas, USA) for 10 min at room temperature, then permeabilized in 0.05% Triton X-100 (Eurobio Scientific, Les Ullis, France) in PBS for 1 h at room temperature before blocking with 2% bovine-serum-albumin (BSA) (Eurobio Scientific) in PBS for 1 h at room temperature to avoid nonspecific binding. M0-MDMs were then stained with 1 µg/mL rabbit anti-LC3B primary antibody (Novus Biologicals, Centennial, USA) in 2% BSA-PBS overnight at 4°C. Cells were finally stained with 2 µg/mL DAPI and 1 µg/mL goat anti-rabbit-Alexa647 secondary antibody (Thermo Fischer Scientific) for 1 h at room temperature. After washings, coverslips were mounted with Dako Fluorescence mounting medium (Dako North America, Carpinteria, USA). Fluorescent-labeled cells were captured with a fluorescence microscope (ZEISS AxioImager M1). LAPosomes are identified as fluorescence recruitment of LC3 around phagosomes containing apoptotic Jurkat cells. At least 10 random quantifications per condition were performed manually and blindly by two independent investigators with the Fiji software and averaged for each condition.

### 2.9 Cell surface receptor analyses by flow cytometry

After cell washing and detachment using Accutase^®^ cell detachment solution (BioLegend, Paris, France), MDMs were stained with Fixable Viability Stain 780 (BD Biosciences, Le Pont de Claix, France) for 10 min at room temperature. MDMs were first blocked in PBS supplemented with 2% FBS solution containing FcR blocking reagent (Miltenyi Biotec SAS) for 10 min at room temperature to avoid nonspecific binding, and then re-suspended and incubated with specific antibodies or appropriate isotype controls for 30 min at 4°C. Cells were washed with PBS, collected by centrifugation (2500 rpm for 5 min) and then analyzed on a LSR II cytometer and FlowLogicTM software (Miltenyi Biotec SAS). The quantification of efferocytosis receptors was performed using the following antibodies: FITC anti-CD36, FITC anti-ITGβ5, PE anti-MERTK (BioLegend), APC anti-CD36L1/SR-B1 (Miltenyi Biotec SAS), and their respective isotype control, as recommended by BD Biosciences (Le Pont de Claix, France). Results were expressed as the mean ratio of median fluorescence intensity (MFI) calculated as follows: MFI (mAb of interest)/MFI (isotype control mAb).

### 2.10 Reverse transcription-quantitative polymerase chain reaction

Total RNA was extracted from human cells and mouse tissue with a Nucleospin RNA extraction Kit (Macherey-Nagel, Hoerdt, France) according to the manufacturer’s instructions. RNA concentrations were measured by spectrofluorimetry using a NanoDrop 1000 (Thermo Fisher Scientific) and reverse transcribed using the High-Capacity cDNA Reverse Transcription Kit (Thermo Fisher Scientific). Quantitative PCR (qPCR) assays were next performed using the fluorescent dye SYBR Green methodology and a CFX384 Real-Time PCR detector (Bio-Rad Laboratories, Marnes-la-Coquette, France). Human and mouse predesigned KiCqStart^®^ SYBR^®^ Green primers for gene expression analysis were purchased from Sigma-Aldrich. The specificity of amplified genes was evaluated using the comparative cycle threshold method (CFX Manager Software). The Mean of Cq values was used to normalize the expression of the steady-state target mRNA levels to those of the 18S ribosomal protein, using the 2(−ΔΔCq) method.

### 2.11 Quantification of cytokine secretion levels

Levels of IL-6, TNFα, CCL18, and CCL22 secreted in MDM culture media were quantified using specific Duoset ELISA kits from R&D Systems, Bio-Techne, France: IL-6: DY206; for TNFα: DY210; for CCL18: DY394; and for CCL22: DY336). Levels of S100A8 in mouse lung extract were also quantified by ELISA (R&D Systems, DY8596-05). Levels of mouse sera or human plasmatic CXCL4 were also quantified by ELISA (R&D Systems, human: DY795, mouse: DY595).

### 2.12 Statistical analysis

Data were presented as means ± standard error on the mean (SEM). Comparison between more than 2 groups was performed by repeated measure analysis of variance (ANOVA) followed by Dunnett’s or Newman–Keuls multiple comparison *post hoc* test. Depending on the conditions, Student’s paired-or unpaired t-tests were used to compare two groups. A p-value < 0.05 was considered significant. Data analyses were performed with GraphPad Prism 5.0 software (GraphPad Software, La Jolla, CA, USA).

## 3 Results

### 3.1 CXCL4-rich plasma may contribute to the lower efferocytosis capacities of human monocyte-derived macrophages

**Figure 1:**
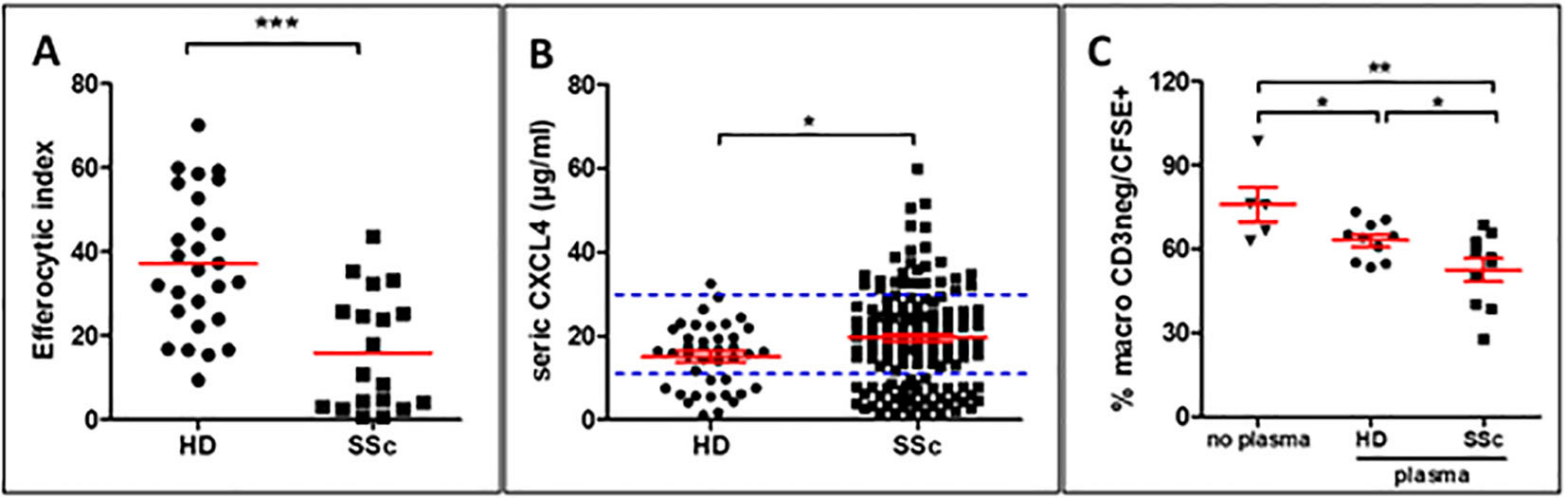
Role of CXCL4-rich plasma on efferocytosis capacities of human monocyte-derived macrophages (MDMs) **(A)** Evaluation of efferocytosis capacities of CFSE^+^ apoptotic Jurkat cells by M0-MDMs from healthy donors (HD) and SSc patients by flow cytometry. The efferocytic index represents the mean ± SEM of HD (n=27) and patients with SSc (n=19). Student unpaired t-test, ***p< 0.001. **(B)** Evaluation of CXCL4 levels (µg/ml) in the serum of HD (n=42) and patients with SSc (n=150). Student unpaired t-test, *p< 0.05. **(C)** Evaluation of efferocytosis capacities of M0-MDMs exposed to HD or SSc plasma. The engulfment of CFSE^+^ apoptotic Jurkat cells by MDMs was expressed as a % of MDMs by flow cytometry after matching on age and sex for both HD and SSc. Data are expressed as mean ± SEM. ANOVA Newman-Keuls multiple comparison *post hoc* test, *p< 0.05; **p< 0.01.

As we previously described, MDMs from patients with SSc have significantly decreased capacities to eliminate apoptotic cells (n=19) when compared to MDMs from healthy donors (HD) (n=27) (**Figure 1A**) (4). We also confirmed that CXCL4 serum levels were significantly higher in SSc patients (n=150) than those of HD (n=42) (**Figure 1B**) (12). The clinical characteristics of patients with SSc in **Figures 1A** and **B** are reported in **Supplementary Tables 1** and **2**, respectively. To evaluate the impact of soluble factors from the serum of SSc patients on the efferocytosis capacities of macrophages, we exposed human MDMs isolated from HD to plasma from HD or SSc patients and then exposed MDMs to CFSE^+^ apoptotic Jurkat cells to compare their phagocytic index. MDMs exposed to HD and SSc plasma showed a significant decrease in efferocytosis when compared to MDMs exposed to a control medium and MDM exposed to SSc plasma patients exhibited a significant decrease in efferocytosis properties when compared to MDM exposed to HD plasma (**Figure 1C**). Altogether, these data strongly suggested that serum elements like soluble CXCL4 may contribute to defect of efferocytosis.

### 3.2 SSc-ILD mouse models induced by either BLM and HOCl exhibit CXCL4-MDMs markers, a phenotype associated with defective efferocytosis function *in vivo*

**Figure 2:**
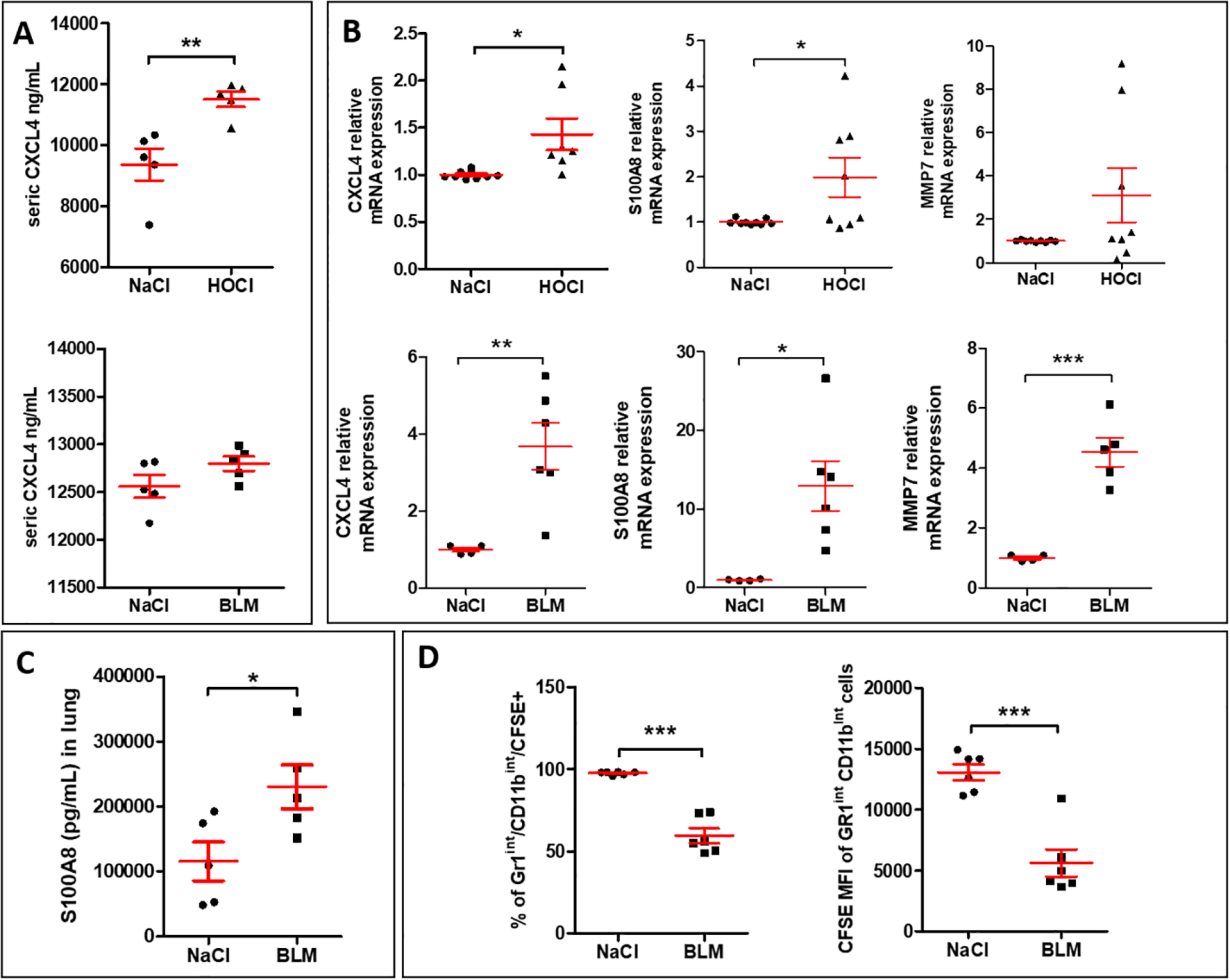
SSc-ILD mouse models induced by either HOCl or BLM exhibit CXCL4-MDM markers, a phenotype associated with defective efferocytosis function *in vivo.* **(A)** Evaluation of CXCL4 secretion levels (pg/ml) by ELISA in the sera of HOCl and BLM mouse models in comparison with the saline (NaCl) control group. All experiments are the results of duplicate experiments and are expressed as means ± SEM (n = 5 mice per group). Student unpaired t-test, **p < 0.01. **(B)** Evaluation of M4 signature in the lung of HOCl and BLM mouse models. The mRNA relative expressions are expressed as means ± SEM with saline group (NaCl) arbitrarily set to 1 (n=4 to 9 mice). Student unpaired t-test, *p< 0.05; **p< 0.01; ***p<0.001. **(C)** Evaluation of S100A8 secretion levels (pg/ml) in lung extracts of BLM mice in comparison with the saline (NaCl) control group by ELISA. The results are expressed as means ± SEM (n = 5 mice per group). Student unpaired t-test, *p< 0.05. **(D)** Evaluation of efferocytosis capacities of murine alveolar macrophages (AM) in BLM mouse model. The percentage of AM (Gr1^Int^ and CD11b^Int^) that have engulfed CFSE^+^ apoptotic Jurkat cells and the median fluorescence intensity (MFI) of CFSE+ AM were presented as the means ± SEM (n = 6 mice per group). Student unpaired t-test, ***p<0.001.

As the expression of the chemokine CXCL4 was previously correlated with the fibrotic complications of the disease (12), we explored its expression in two mouse models of SSc-ILD induced by intradermal injections of repeated BLM or HOCl in which lung fibrosis has been validated (18, 19). In the HOCl model, serum levels of CXCL4 were significantly elevated when compared to the saline (NaCl) group, while in the BLM model, CXCL4 levels showed a moderate increase that did not reach statistical significance (**Figure 2A**). The mRNA expressions of *CXCL4* in lung tissues were significantly increased in both BLM- and HOCl-treated mice. Such inductions were accompanied by a significant overexpression of some M4 macrophagic markers such as *S100A8* and *MMP7*, with a greater induction in the lung of BLM-mice as *MMP7* mRNA overexpression was not significant in HOCl-treated mice (**Figure 2B**). Protein expression of S100A8 in the lung was also significantly increased in BLM-exposed mice (**Figure 2C**). To evaluate whether the presence of an M4 macrophage phenotype was associated with an efferocytosis defect, we administered CFSE^+^ apoptotic Jurkat cells to NaCl- or BLM-treated mice. Our results demonstrated that the percentage of AMs (Gr1^Int^ and CD11b^Int^) that ingested CFSE^+^ apoptotic Jurkat cells and the fluorescence intensity of CFSE per macrophage were both significantly decreased in the BAL of BLM-induced SSc mice when compared to the control group (**Figure 2D**). Altogether, these results strongly suggested that SSc-ILD mice presented at the systemic and the lung tissue levels a M4 phenotype and demonstrated that AMs have a defect of efferocytosis.

### 3.3 CXCL4-induced M4-MDMs exhibit a mixed polarized phenotype and a defect of phagocytosis and efferocytosis capacities

**Figure 3:**
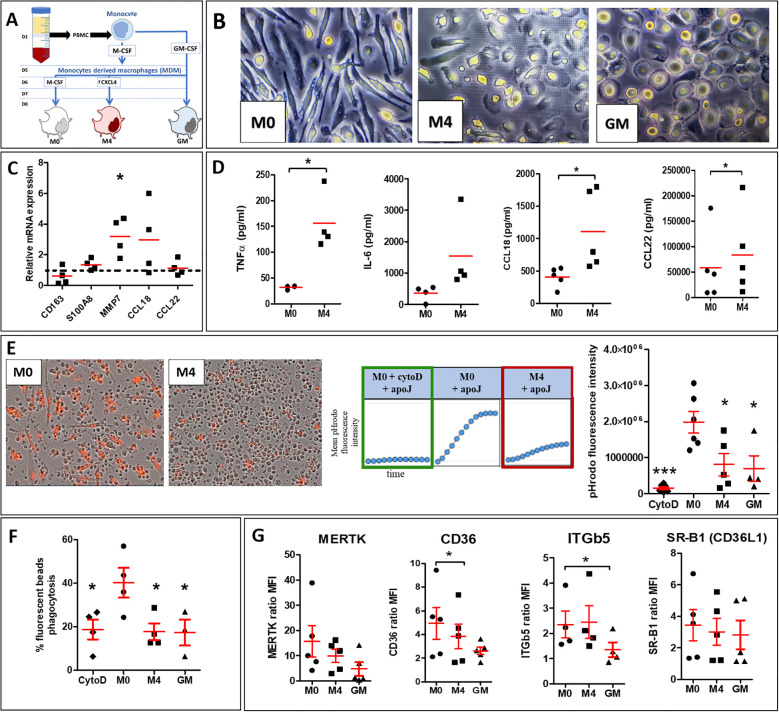
Phenotype and functions of CXCL4-induced M4-MDMs. **(A)** Simplified schema summarizing the steps to follow to obtain polarized human MDMs from healthy donors (HD). **(B)** Morphology of human MDMs (M0, M4, and GM) observed by light microscopy at x20 magnification. **(C)** Evaluation of mRNA expression by RT-qPCR in M4-MDMs relative to control resting M0-MDMs, arbitrarily set to 1 unit (dashed line). All experiments are the results of duplicate experiments conducted in MDMs from n = 4 to 6 independent HDs and are expressed as means ± SEM. Student paired t-test, *p< 0.05. **(D)** Evaluation of cytokine secretion levels (pg/ml) in the culture medium of M4-MDMs relative to control resting M0-MDMs by ELISA. All experiments are the results of duplicate experiments conducted in MDMs from n = 4 to 6 independent HDs and are expressed as means ± SEM. Student paired t-test, *p< 0.05. **(F)** Evaluation of phagocytic capacities of M4 by flow cytometry (see methods) relative to control resting M0-MDMs. Data are expressed as mean % of phagocytosis (ratio of median fluorescence intensity (MFI) at 37°C/MFI at 4°C) ± SEM from 4 independent HDs, ANOVA Dunnett’s *post hoc* test compared to resting M0-MDMs, * p< 0.05. **(E)** Evaluation of efferocytosis capacities of pHrodo^+^ apoptotic Jurkat cells by M4-MDMs compared to resting M0-MDMs pre-treated or not with CytoD. Data are expressed as mean pHrodo fluorescence intensity ± SEM of 4 independent experiences in duplicate, ANOVA Dunnett’s *post hoc* test compared to resting M0-MDMs. *p< 0.05; ***p< 0.001. **(G)** Expression of cell surface receptor involved in efferocytosis in M4-MDMs by flow cytometry relative to control resting M0-MDMs. Data are expressed as mean fluorescence intensity (MFI) relative to isotype control (ratio) ± SEM, from 5 independent HDs, Student paired t-test, *p< 0.05.

To better understand the effects of CXCL4 on MDM efferocytosis capacities, we characterized human M4-MDMs functions *in vitro* and explored their phenotype as compared to previous descriptions (15) (**Figure 3A**). Microscopic observation of MDMs in culture revealed that M4-MDMs had less spindles than M0-MDMs and they were rounded, showing similarities with GM-CSF-differentiated MDMs (GM-MDMs) (**Figure 3B**). Among the M4 markers previously identified (20), we found a significant increase of MMP7 mRNA expression in comparison to M0-MDMs, whereas CD163 mRNA expression was reduced in M4-MDMs, without reaching statistical significance. The mRNA expression of S100A8 and the M2a/M4 markers, CCL18 and CCL22 were found unchanged when compared to M0-MDMs (**Figure 3C**). Flow cytometry analysis of membrane markers showed that M4-MDMs tend to have reduced expression of CD163, that was previously found downregulated in M4 macrophages (21), and of CD86, an M1-marker when compared to M0-MDMs whereas the expression of CD206, a M2a marker, was found unchanged (**Supplementary Figure 1**). M4-MDMs also showed a pro-inflammatory phenotype with a high secretion of cytokines such as IL-6 and TNF-α as compared to M0-MDMs. Interestingly, M4-MDMs also secreted higher levels of the pro-fibrotic M2 markers CCL18 and CCL22 as compared to M0-MDMs (**Figure 3D**). Altogether, our results suggest a specific phenotype of CXCL4-induced M4-MDMs, with mixed pro-inflammatory and pro-fibrotic features.

We secondly evaluated the phagocytic and efferocytic functions of M4-MDMs. The capacities of M4-MDMs to phagocyte fluorescent pHrodo+ apoptotic cells, determined by real-time fluorescence microscopy, were significantly reduced. GM-MDMs and M0-MDMs pretreated with cytochalasin D (cytoD), a drug inhibiting phagocytosis via the impairment of actin depolymerization and cytoskeleton remodeling (22), also showed decreased efferocytosis capacities (**Figure 3E**). By flow cytometry, we showed that M4-MDMs were also less efficient in engulfing FITC-beads than M0-MDMs and that their phagocytic abilities were similar to those of GM-MDMs and MDMs exposed to cytoD (**Figure 3F**). To better understand such an alteration of efferocytosis in CXCL4-induced MDMs, we evaluated by flow cytometry the membrane expression of several receptors involved in the recognition of apoptotic cells. As previously described (23), the expression of CD36 was significantly reduced in M4-MDMs in comparison to M0-MDMs. The expression of the CD36L1 and MERTK also tended to decrease in M4-MDMs but without reaching statistical significance whereas ITGβ5 expression did not differ between M4-MDMs and M0-MDMs (**Figure 3G**). Altogether, these results suggested that the moderate decrease in CD36 expression could not fully explain the reduced efferocytosis capacities of M4-MDMs and that other mechanisms could be involved.

### 3.4 CXCL4-induced M4-MDMs show enhanced LC3-associated phagocytosis

**Figure 4:**
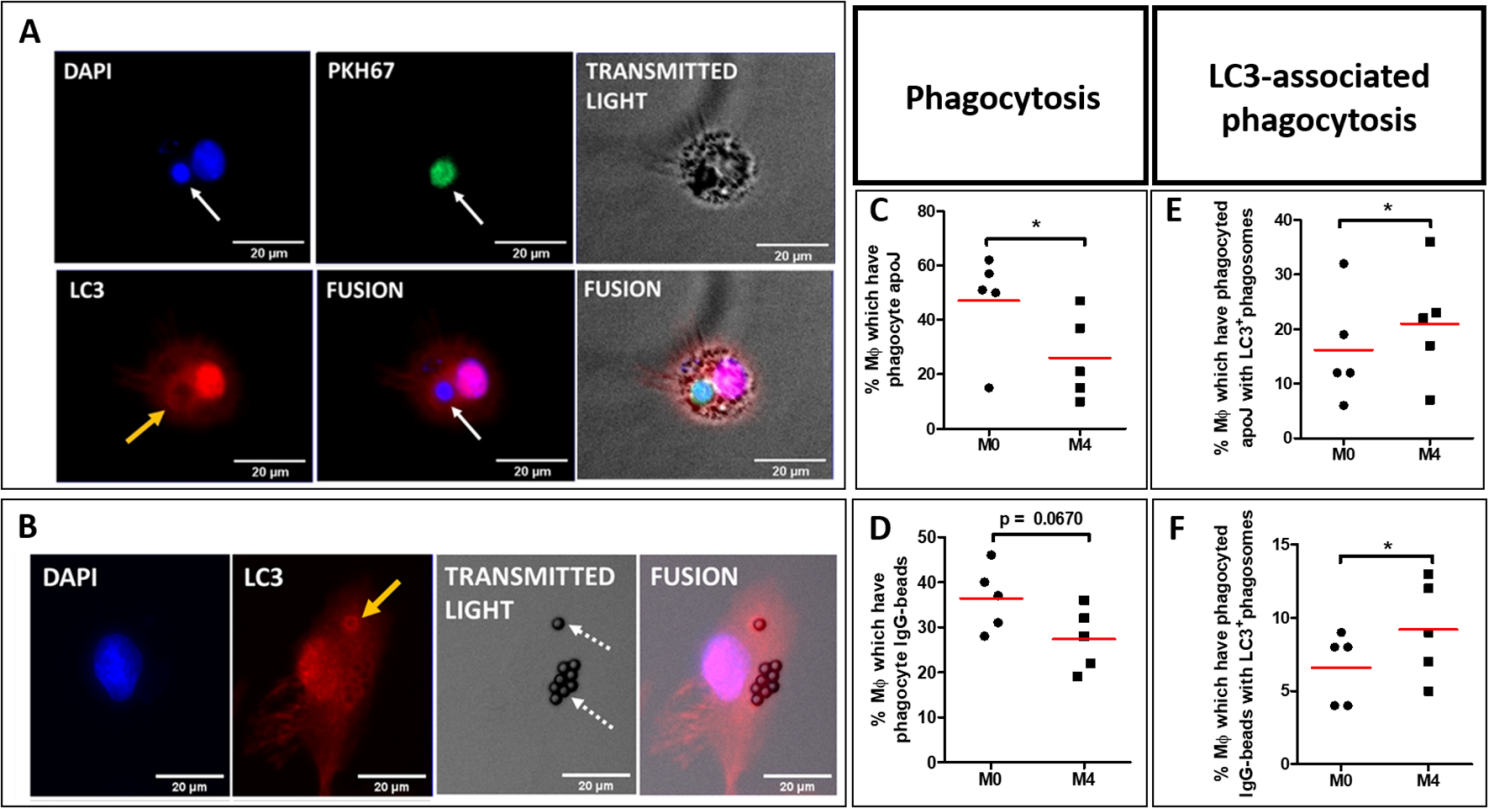
LAPosome quantification in polarized MDM from healthy donors (HD). **(A, B)** M0 and M4 were primarily exposed to either PKH67-stained (green) apoptotic Jurkat cells (PKH67+ apoJ) (white arrow, **A**), or IgG-coated beads (dotted arrows, **B**) for 45 min. Nuclei were then stained with DAPI (blue), and LC3 stained with rabbit anti-LC3B primary antibody targeted with goat anti-rabbit Alexa647 secondary antibody (red). LC3+ phagosomes (LAPosomes) are characterized by circular red fluorescence recruitment around phagosomes (yellow arrows). **(C, D)** Evaluation of phagocytosis capacities of M4-MDMs relative to M0-MDMs were obtained from 5 independent HDs, and not 4. **(C)** or IgG-coated beads **(D)** after 45 min of phagocytosis. Phagocytosis data represent the mean ± SEM of the percentage of MDMs that have phagocytosed either at least one PKH67+ apoptotic Jurkat cells (apoJ) **(C)**, or at least one IgG-coated bead **(D)**, from 4 independent HDs. Ten random quantifications per condition were performed manually and blindly by two independent investigators with the Fiji software and averaged for each condition. Student paired t-test, *p < 0.05. **(E, F)** Evaluation of LC3-associated phagocytosis (LAP) capacities of M4-MDMs relative to M0-MDMs. LAPosomes are identified as fluorescence recruitment of LC3 around phagosomes in cells containing either PKH67+ apoptotic Jurkat cells (apoJ) **(E)** or IgG-coated beads **(F)** after 45 min of phagocytosis, as described in **(A, B)**, respectively. LAP data represent the mean ± SEM of the percentage of MDMs showing at least one LC3+ phagosome, among MDMs that have phagocytosed either PKH67+ apoJ **(E)** or IgG-coated beads **(F)**, from 5 independent HDs. Ten random quantifications per condition were performed manually and blindly by two independent investigators with the Fiji software and averaged for each condition. Student paired t-test, *p < 0.05.

To explain the lower abilities of M4-MDMs to accumulate apoptotic cells in phagolysosomes (**Figure 3E**), we explored the LC3-associated phagocytosis (LAP), an internalization process utilizing some proteins of the autophagy machinery. LC3 recruitment and conversion are involved in the canonical and non-classical autophagy pathways. It is well known that the recruitment of LC3 relies on the formation and elongation of the phagophore which is slow during autophagy unlike the LAP process characterized by a quick LC3 recruitment that is performed in less than 15 min after apoptotic cell exposure and that reaches a maximum after one hour (24, 25). M4-MDMs were thus exposed to PKH67^+^ apoptotic Jurkat cells for 45 min before cellular immunostaining of LC3, and LAPosome quantification by fluorescence microscopy. LAPosomes are characterized by circular recruitment of the LC3 protein surrounding the apoptotic Jurkat cells (**Figure 4A**), or IgG-beads (**Figure 4B**), as opposed to phagosomes without peri-phagosomal reinforcement of LC3 staining. While we confirmed the decreased phagocytic capacities of CXCL4-induced MDMs to engulf apoptotic Jurkat cells (**Figure 4C**) and IgG-beads (**Figure 4D**) in comparison to resting M0-MDMs, LAPosome proportion among phagosomes was found to be higher in M4-MDMs as compared to M0-MDMs after a 45-minute efferocytosis of apoptotic Jurkat cells (**Figure 4E**) or of IgG-beads (**Figure 4F**). LAPosomes only represented a moderate proportion of the total number of phagosomes, LAP being a dynamic and fleeting process. MDMs also showed a diffuse cytoplasmic and nuclear fluorescence of LC3, the protein being mainly in its soluble form LC3-I with a nuclear reservoir (26).

**Figure 5:**
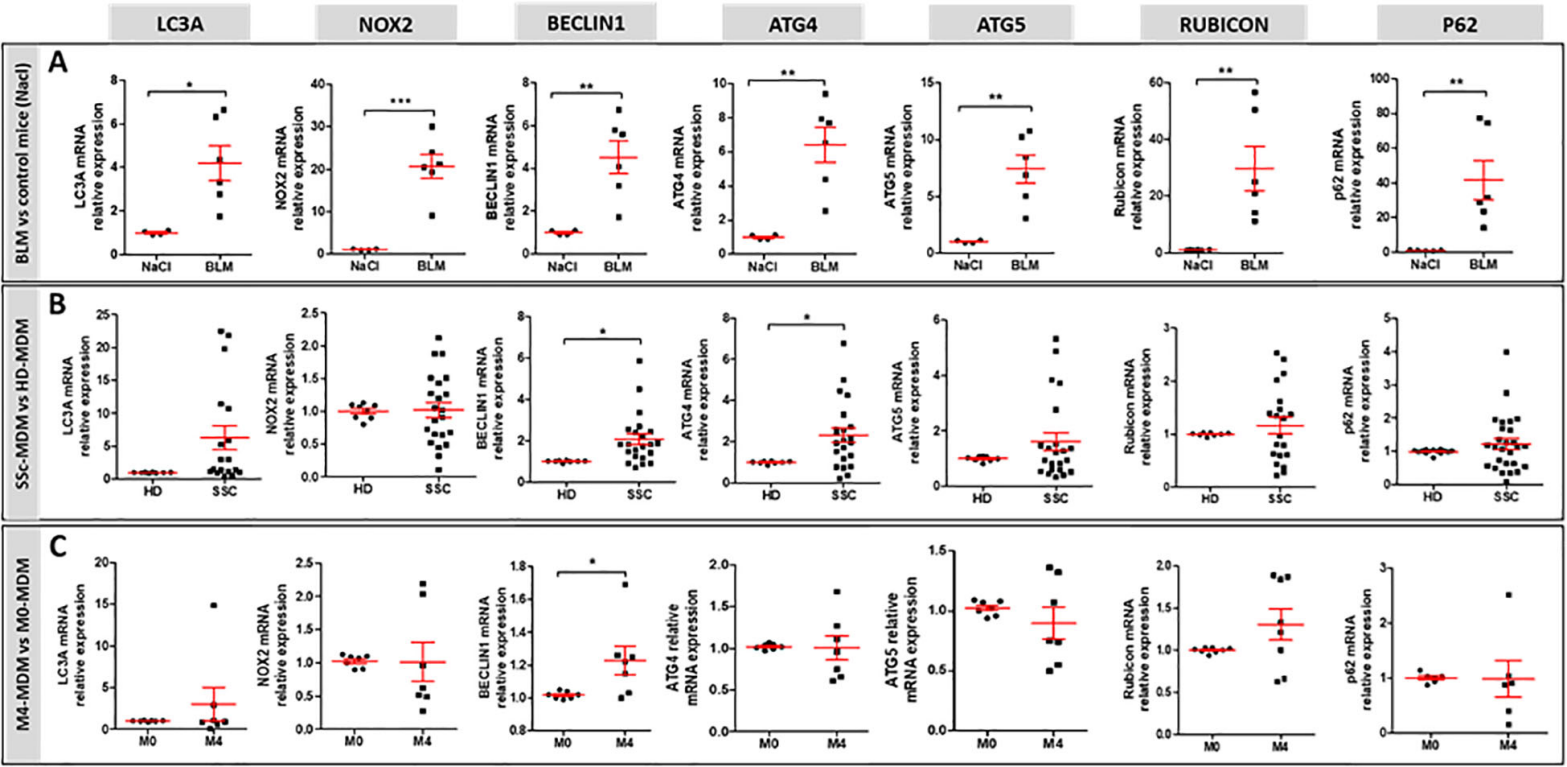
mRNA expression of genes involved in LAP. **(A)** mRNA expression in the lung of BLM-exposed mice (n=6 mice) compared to saline group (n=4 to 6). Student unpaired t-test, *p< 0.05; **p< 0.01; ***p<0.001. **(B)** mRNA expression in SSc-MDMs compared to HD-MDMs. All experiments are the results of duplicate experiments conducted in MDMs from independent HDs (n=8 to 14) and SSc patients (n=19 to 27). Student unpaired t-test, *p< 0.05. **(C)** mRNA expression in M4-MDM compared to M0-MDMs. All experiments are the results of duplicate experiments conducted in MDMs from n = 6 to 8 independent HDs and are expressed as means ± SEM. Student paired t-test, *p< 0.05.

To explain the higher LAPosome proportion in M4-MDMs, we next evaluated the expression of autophagy-associated proteins involved in LAP both *in vivo* and *in vitro*. We analyzed Rubicon and NOX2 mRNA expression, both known to promote LAP. These proteins are part of a complex that also includes BECLIN1. Additionally, we analyzed the mRNA expression of the ATG (autophagy-related) proteins ATG4 participating in the conversion of LC3-I to its conjugated form LC3-II, and ATG5 involved in LC3-II integration into the phagosome to form LAPosomes. mRNA expression of *LC3A*, *ATG4*, *ATG5*, *BECLIN1*, *NOX2*, *Rubicon*, and *p62* were all significantly increased in the lung of BLM-SSc mice in comparison to controls (**Figure 5A**). By contrast, only *BECLIN1* and *ATG4* mRNA expression were upregulated in SSc-MDMs when compared to HD-MDMs (**Figure 5B**), and only *BECLIN1* in M4-MDMs compared to M0-MDMs (**Figure 5C**). The clinical characteristics of patients with SSc of **Figure 5B** are reported in **Supplementary Table 1**. Altogether, our data suggest that, while phagocytosis was less efficient, LAP seemed increased *in vivo* in the fibrotic lungs of BLM-mice as well as *in vitro* in SSc-MDMs, with enhanced LAP in M4-MDMs *in vitro*.

## 4 Discussion

In this study, we demonstrated that CXCL4 participates in the alteration of efferocytosis in SSc through the induction of a specific M4 macrophage phenotype. To our knowledge, this is the first study investigating the role of this particular macrophage subtype in SSc pathogenesis in human and in SSc mouse models.

*In vivo*, our results are consistent with those of Affandi et al., who observed increased CXCL4 serum levels and mRNA expression in the skin of a BLM-mice model of fibrosis, correlating CXCL4 expression with skin and lung fibrosis (27). In our study, we demonstrated that efferocytosis alteration of AMs in the BLM-mice model could be associated with the upregulation of both *CXCL4* and the two key M4 macrophage markers *MMP7* and *S100A8* (28) in the lungs. This finding is supported by Zuo et al., who previously identified MMP7 as a mediator of pulmonary fibrosis in response to BLM (29). Furthermore, we confirmed the potential implications of CXCL4-driven macrophages in fibrotic lungs using a second SSc mouse model induced by repeated injection of HOCl. Interestingly, previous reports have also described elevated expression of MMP7 (30) and S100A8 (31) in patients with SSc, both of which were correlated with the severity of pulmonary fibrosis. Additionally, Piguet et al. demonstrated that BLM instillation in mice increased both collagen deposition and platelet trapping within alveolar capillaries (32). Heparin treatment significantly reduced BLM-induced lung fibrosis without decreasing the number of platelets, suggesting a potential role of platelets mediators such as CXCL4, while it has been described that heparin specifically binds and inhibits CXCL4 effects on monocytes (21, 33).

*In vitro*, we demonstrated that M4-MDMs expressed some pro-fibrotic markers associated with the M2 polarization profile (CCL18 and CCL22 secretions), but also had a pro-inflammatory phenotype with impaired phagocytosis and efferocytosis capacities similar to GM-MDMs. This dual pro-inflammatory and pro-fibrotic phenotype aligns with the concept of a mixed signature of macrophages involved in SSc, beyond the M1/M2 dichotomy (9, 10). M4-MDMs markers in our study are consistent with those from previous studies (15, 20, 23). However, variations in the expression of some markers can be explained by differences in experimental conditions. For example, the loss of the CD163 receptor at the membrane, reported as M4 specific by some authors (21), was only moderate in our study; such discrepancies could be explained by lower concentrations of CXCL4 used in our study and a shorter exposure time (2 days versus 6 days), which were chosen to facilitate comparison between different MDM profiles. The co-exposure to CXCL4 and M-CSF may have also influenced the polarization of M4-MDM. However, another work using a polarization protocol similar to ours also reported minor differences between the transcriptomes of M0- and M4-MDMs (34).

Despite these few phenotypic differences, a moderate exposure to CXCL4 significantly impaired MDM functions, with significantly reduced phagocytosis and efferocytosis capacities. Atherosclerosis, another fibroproliferative and inflammatory disease, is also characterized by impaired efferocytosis linked to a reduced expression of efferocytosis-related receptors such as MERTK and CD36 (35, 36). Additionally, the genetic deletion of *CXCL4* has been shown to decrease atherosclerosis (37) and exogenous CXCL4 has been observed to diminish the phagocytic capacities of macrophages involved in myocardial infarction by reducing the expression of CD36, a dual receptor involved in both the engulfment of apoptotic cells and the uptake of modified lipids (23). In our study, CD36 receptor expression was also decreased, although such moderate decrease could not explain the major efferocytosis defect of M4-MDMs. However, altered efferocytosis in M4-MDMs could be attributed to a significant reduction in phagocytic function. The dysfunction of this process could be explained by the activation of the RhoA/ROCK signaling pathway, which is involved in cytoskeletal remodeling and phagocytosis (38). This signaling pathway is activated by exposure to crystalline silica, an environmental risk factor of SSc, and is implicated in the phenotypic changes of SSc-MDMs (39). Additionally, in the pro-inflammatory GM-MDM phenotype, which is morphologically similar to M4-MDMs, the RhoA/ROCK pathway is also activated (40). Therefore, their round shape observed under optical microscopy, in contrast to the fusiform aspect of M0- and M2-MDMs, may reflect reduced deformability of the cellular cytoskeleton, limiting phagocytic capacities.

While M4-MDMs exhibited low phagocytosis capacities *in vitro*, we found an increase in LAPosomes proportion among phagosomes compared to resting M0-MDMs. However, we did not observe an overexpression of autophagic genes in M4-MDMs, except for *BECLIN1*, when compared to resting M0-MDMs. As M4-MDMs are less efficient to engulf apoptotic cells, and because LAP is a dynamic process, it is also possible that LC3-phagosome conjugation has already occurred in M0-MDM, thus being less visualized as compared to an earlier uptake. Therefore, an early analysis of LAP machinery early after phagocytosis and at the protein level would be more appropriate to explain the increase of LAPosome proportion. An increased lysosomal degradation could also explain this increase of LAPosomes in M4-MDMs. Intriguingly, a previous study showed that LAP was enhanced in circulating monocytes from patients with hepatic cirrhosis and in a mouse model of liver fibrosis and that LAP inhibition led to increased hepatic inflammation and fibrosis, suggesting that LAP could act as a compensatory mechanism preventing MDMs reprogramming toward a proinflammatory phenotype (41). In our study, we found an increase in autophagic gene expression in the lungs of BLM-SSc mice. These results notably align with those of Mori et al. and Frech et al. showing a higher expression of LC3 by immunofluorescence in the lesional dermis of both BLM-treated mice and SSc patients compared to controls (42, 43). Moreover, Cabrera et al. found that *ATG4* gene expression was significantly increased in BLM-treated mice, thus preventing lung apoptosis and increasing inflammatory response (44). As mRNA expression was evaluated on total lung extract in our study, beyond macrophages, autophagy from other cell types (lung fibroblasts, lung epithelial cells) could explain these results. In SSc-MDMs, despite showing a significantly higher expression of *BECLIN1* and *ATG4*, autophagic gene expression was more heterogeneous, which is consistent with the heterogeneity of MDMs phenotypes in this disease.

Previous reports argue for a connection between autophagy machinery and fibrosis (45). Autophagy machinery was activated in SSc fibrotic skin in a TGFβ-dependent manner in previous studies, thereby promoting collagen release through BECLIN1 overexpression (46). Moreover, Liu et al. demonstrated that hypoxia enhanced autophagic protein expression concomitant with fibroblast collagen synthesis (47). As platelets are a significant source of TGFβ and CXCL4, both have been found elevated in SSc patients with Raynaud’s phenomenon (48, 49). Given that vasculopathy is an early event in SSc pathogenesis, endothelial damage occurring during the initial stages of the disease may activate platelets, leading to subsequent release of mediators such as CXCL4 (50), linking autoimmunity and fibrosis by its effects on monocytes (51, 52).

There are several limitations to our study, including the inability to establish the presence of a specific M4 macrophage population among human SSc-MDMs. Additionally, we did not correlate the altered level of efferocytosis of AMs *in vivo* in SSc mice with the expression level of *CXCL4* in the lungs. Despite our exploration of LAP for a better understanding of efferocytosis alteration in SSc- and M4-MDMs, we cannot correlate both processes. Finally, the absence of difference in the autophagic gene expression between M4- and M0-MDMs might be attributed to a lack of statistical power due to the limited sample size of the groups. Nevertheless, we have shown that the phagocytosis capacities of human MDMs can be influenced by the extracellular environment, with significant impairment of efferocytosis by CXCL4. Although we did not identify the specific CXCL4 receptor responsible for these effects, CCR1 or chondroitin sulfate proteoglycan receptors are strong candidates (21). Exposure to CXCL4 induced a specific M4 macrophage phenotype characterized by a mixed proinflammatory and profibrotic profile. Our study thus strengthens the role of CXCL4 in the pathogenesis of SSc, providing additional evidence of a connection between vasculopathy, autoimmunity, and fibrosis in this severe autoimmune disorder.

## Data Availability

The original contributions presented in the study are included in the article/**Supplementary Material**. Further inquiries can be directed to the corresponding author.
