# Supplementary material for: Efferocytosis dysfunction in CXCL4-induced M4 macrophages: phenotypic insights in systemic sclerosis in vitro and in vivo
Source: Front Immunol. 2024 Oct 11;15:1468821. doi: 10.3389/fimmu.2024.1468821 (PMC11512447; doi:10.3389/fimmu.2024.1468821)
Supplement: Supplementary file 1 [file DataSheet1.docx]

**Efferocytosis dysfunction in CXCL4-induced M4 macrophages: phenotypic insights in systemic sclerosis in vitro and in vivo

Efferocytosis Dysfunction of M4 Macrophages**

Erwan Le Tallec^a, b^ ^+¶^, Nessrine Bellamri ^a +¶^, Marie Lelong ^a^, Claudie Morzadec ^a^, Quentin Frenger^c, d^,
Alice Ballerie ^a, b^, Claire Cazalets^b^, Alain Lescoat^a, b^, Frédéric Gros ^c, d^, Valérie Lecureur^a*^

**SUPPLEMENTARY FIGURES**

**
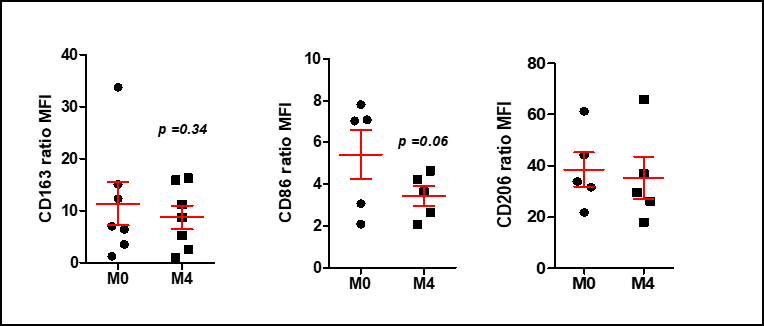
**

**Supplementary Figure 1: Membrane expressions of polarized macrophagic markers in M4-MDMs by flow cytometry**.

Expression of polarization markers of monocyte-derived macrophages (MDMs) in M4-MDMs. MDMs were polarized for 48 h by adding 1 µM of CXCL4 or unpolarized (M0). Cells were harvested stained and the expression of cell surface molecules was analyzed by flow cytometry. Data are expressed as the mean ratio of MFI + SEM for at least 5 independent experiments.


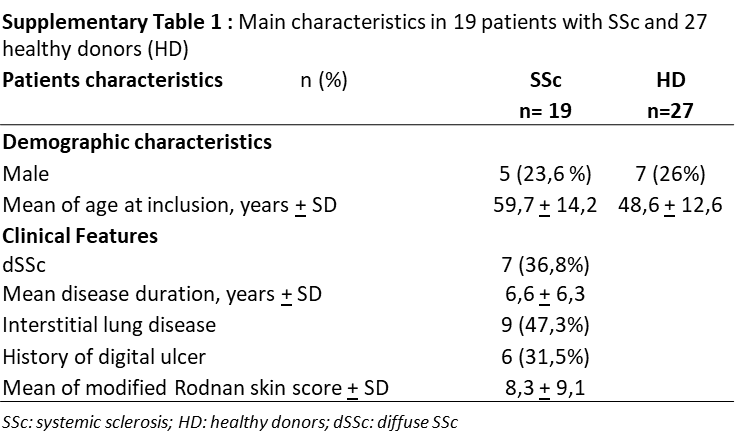
**Supplementary Table 1: Main characteristics in 19 patients with systemic sclerosis (SSc) and 27 healthy donors (HD)**

**
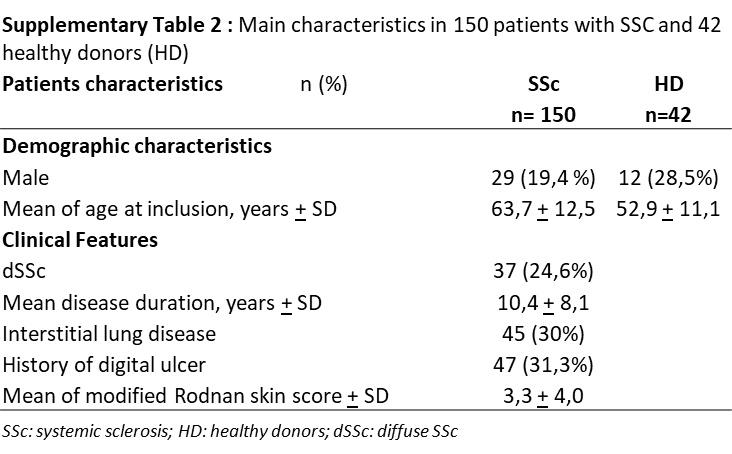
Supplementary Table 2: Main characteristics in 150 patients with systemic sclerosis (SSc) and 42 healthy donors (HD)**
